# On the Number of Species Required to Detect Stabilizing Selection in Gene Expression Evolution

**DOI:** 10.1093/gbe/evag266

**Published:** 2026-10-24

**Authors:** Haoqing Du, Nicolás Lichilín, Ana Catalán, Sebastian Höhna

**Affiliations:** Department of Earth and Environmental Sciences, Paleontology & Geobiology, Ludwig-Maximilians-Universität München, München 80333, Germany; GeoBio-Center, Ludwig-Maximilians-Universität München, München 80333, Germany; Division of Evolutionary Biology, Ludwig-Maximilians-Universität München, München 82152, Germany; Division of Evolutionary Biology, Ludwig-Maximilians-Universität München, München 82152, Germany; Department of Earth and Environmental Sciences, Paleontology & Geobiology, Ludwig-Maximilians-Universität München, München 80333, Germany; GeoBio-Center, Ludwig-Maximilians-Universität München, München 80333, Germany

**Keywords:** phylogeny, Ornstein–Uhlenbeck, Brownian motion, model selection

## Abstract

Differentiating between genes that evolve under neutral drift versus stabilizing selection is one of the first explorations in comparative phylogenomic studies. Neutral drift and stabilizing selection of gene expression levels across a phylogeny are modeled using a Brownian motion and an Ornstein–Uhlenbeck process, respectively. Most empirical gene expression datasets contain only a few species, raising concerns about statistical power for model selection and parameter estimation. Furthermore, gene expression datasets often contain multiple samples per species, contrary to common macroevolutionary analyses of trait evolution. Here, we designed a targeted simulation study to evaluate the power to identify stabilizing selection, emphasizing the impact of the number of species, strength of selection and within-species variances. Our results show that the accuracy of selection strength estimation improves with increasing phylogeny size. Distinguishing weak selection, i.e., lower selection strength, from neutral drift is challenging, whereas varying levels of within-species variance have only minor effects on parameter estimation and model selection. Overall, our results suggest that we can have sufficient power to detect stabilizing selection with approximately 17 species, if the underlying strength of selection is sufficiently strong and if a relatively relaxed statistical significance threshold is adopted.

Significance Phylogenetic models are often used to study how gene expression evolves across species, but it remains unclear whether the datasets with limited number of species provide enough statistical power to detect stabilizing selection across phylogenetic time scales. Compared to macroevolutionary studies of trait evolution, gene expression datasets contain multiple samples per species, adding another layer of complexity. Using empirically guided simulations, we found that the ability to detect stabilizing selection depends mainly on the underlying selection strength and the number of species included in the analysis but not on the amount of within-species variance. Our results provide practical guidance for study design in gene expression evolution across a phylogeny and suggest that datasets must have a sufficient number of species to detect strong stabilizing selection; conclusions might lack power and be biased otherwise.

## Introduction

Divergence in gene expression has long been recognized as a major contributor to phenotypic diversity among species (King and Wilson 1975; Wang et al. 1996). On a macroevolutionary scale, gene expression evolution is argued to be shaped primarily by selection (Gilad et al. 2006; Fay and Wittkopp 2008; Bedford and Hartl 2009; Kalinka et al. 2010; Brawand et al. 2011; El Taher et al. 2021; Wang et al. 2025; Schuster et al. 2026), while other studies suggested that it is dominated by neutral drift (Khaitovich et al. 2004, 2005; Yanai et al. 2004; Catalán et al. 2019; Munro et al. 2022). Two phylogenetic models were introduced to address this debate. The Brownian motion (BM) process describes gene expression evolution under neutral drift (Felsenstein 1985), whereas the Ornstein–Uhlenbeck (OU) process models gene expression evolution under selection, by adding attraction toward an optimal value (Hansen 1997; Butler and King 2004). Particularly, the OU process with a single optimum is typically interpreted as stabilizing selection, while extensions allowing multiple optima are applied to describe directional selection (Hansen 1997; Butler and King 2004; Beaulieu et al. 2012).

The phylogenetic models used for studying gene expression evolution were originally developed for macroevolutionary studies of continuous trait evolution (Bedford and Hartl 2009; Rohlfs et al. 2014; Rohlfs and Nielsen 2015; Catalán et al. 2019; Bertram et al. 2023; Dimayacyac et al. 2023; Pal et al. 2023). Contrary to datasets of continuous traits across a phylogeny, gene expression datasets differ in that they typically contain multiple biological replicates per species (Fig. 1, Table S1), allowing within-species variances to be explicitly incorporated into phylogenetic models. Because within-species gene expression can differ substantially among genes and species, accounting for this variation may influence both parameter estimation and model selection (Price et al. 2022). Although several methods have incorporated within-species variance into models of gene expression evolution (Rohlfs et al. 2014; Catalán et al. 2019; Pal et al. 2023), the extent to which different levels of within-species variation affect evolutionary inference remains poorly understood.

**Fig. 1.**
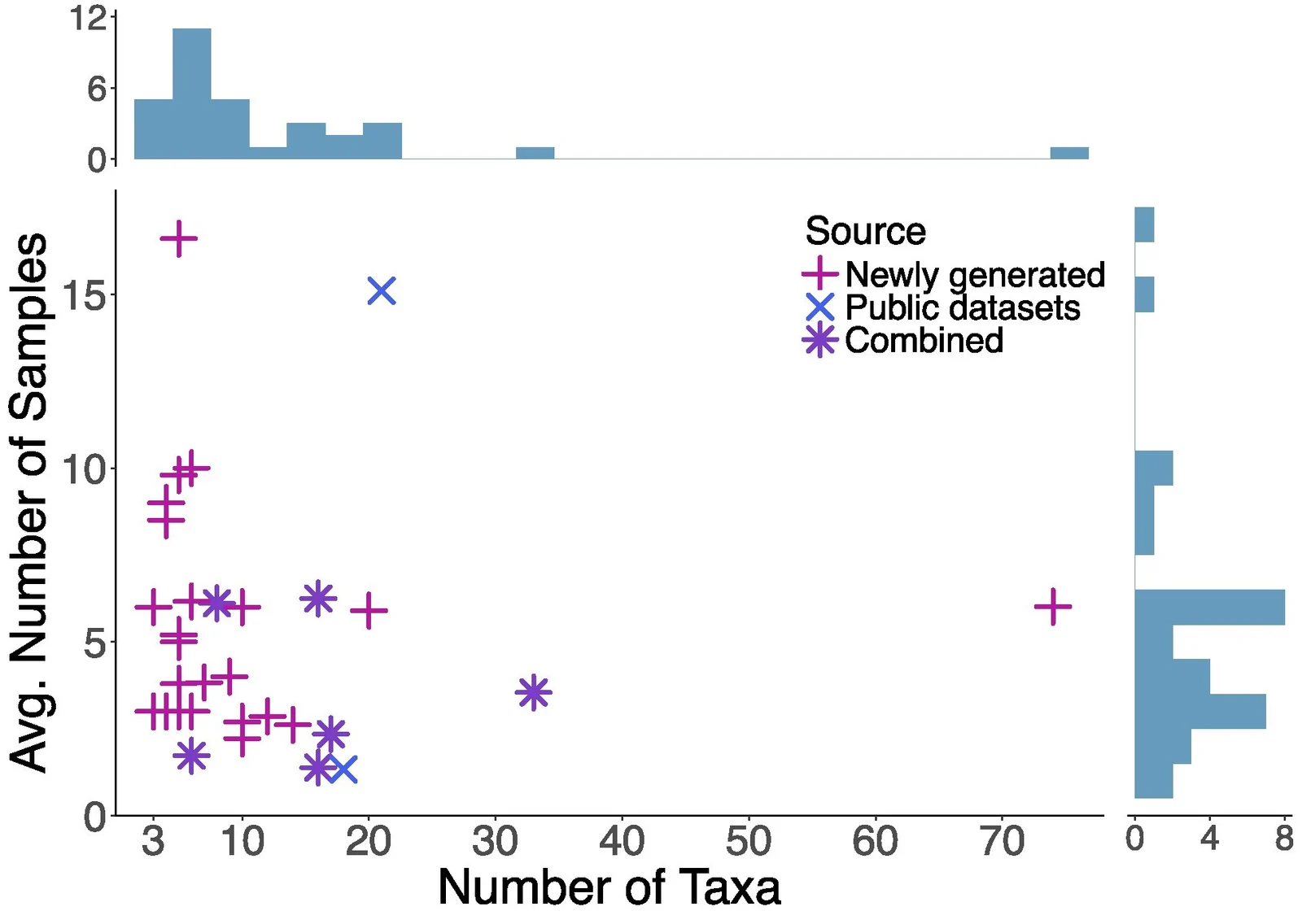
Summary of previous comparative gene expression studies. We collected 33 comparative gene expression studies including data from at least three taxa. The dot plot shows the number of taxa (*x*-axis) and the average number of samples per taxon (*y*-axis) in these studies. The shapes indicate the source of expression data: newly generated datasets (+), publicly available datasets (×), or a combination of newly generated and public datasets ($\convolution$). The marginal histograms show the distributions of the number of taxa and average number of samples per taxon across studies.

Several software packages implement variants of the BM or OU process to study gene expression evolution across a phylogeny (Bedford and Hartl 2009; Rohlfs et al. 2014; Rohlfs and Nielsen 2015; Catalán et al. 2019; Yang et al. 2019; Bertram et al. 2023; Pal et al. 2023). The most common approach infers the dominant mode of gene expression evolution through statistical model selection between BM and OU processes (Kalinka et al. 2010; Catalán et al. 2019; Dimayacyac et al. 2023; Pal et al. 2023; Wang et al. 2025; Schuster et al. 2026). The ability to answer whether gene expression evolution is primarily driven by neutral drift or stabilizing selection depends on several factors: (i) the selection strength, as weak selection might be harder to distinguish from neutral drift; (ii) the number of species (phylogeny size), on which the statistical power fundamentally relies; (iii) the within-species variance, which can propagate uncertainty for the species-mean estimates; and possibly (iv) the statistical approach (e.g., Bayesian inference or maximum likelihood estimation) and the chosen significance threshold.

Among these factors, the phylogeny size is particularly critical for the statistical power of model selection, which for gene expression evolution is the number of species (Bedford and Hartl 2009; Rohlfs et al. 2014; Catalán et al. 2019; Pal et al. 2023). Unlike many macroevolutionary studies of continuous traits, analyses of gene expression evolution are often limited by taxon sampling, as most currently available datasets contain expression data for fewer than 20 species (Fig. 1, Table S1). This limitation raises the question of whether such datasets provide sufficient power to identify stabilizing selection (Catalán et al. 2019; Pal et al. 2023), particularly given evidence that model selection may be biased when phylogenies are small (Boettiger et al. 2012; Cooper et al. 2016; Grabowski et al. 2023; Roa Lozano et al. 2026). Previous work has debated whether as few as five species are sufficient (Pal et al. 2023) or if at least 20 species are required (Catalán et al. 2019). Furthermore, within-species variance, inducing uncertainty of the species-mean gene expression (Ives et al. 2007), could decrease power to correctly detect stabilizing selection. Therefore, it is crucial to quantify the phylogeny size required to differentiate stabilizing selection from neutral drift.

We performed a simulation study guided by empirical choices, i.e. primarily how many and which species to sample, while including a range of selection strengths and within-species variances. Our study assessed whether stabilizing selection can be reliably inferred from gene expression data, in the software RevBayes (v1.3.0; Höhna et al. 2016) and the R Package EvoGeneX (Commit *ece6b07*; Pal et al. 2023), respectively, highlighting the effects of phylogeny size and within-species variance on model selection and parameter estimation.

## Results

### Recovering the Selection Strength *α*

We simulated datasets under four evolutionary models: a BM model and three OU models, each combining with different phylogeny sizes and within-species variances. We then performed Bayesian inference and estimated parameters under an OU model in RevBayes. Our results demonstrated that inference of the selection strength α strongly depends on the phylogeny size. We found that, when the phylogeny size is small, then the posterior distribution of α is strongly influenced by the prior. The posterior distribution increasingly concentrates around the true simulated value as the phylogeny size increases (Fig. 2), and the posterior median value of α becomes more accurate (Fig. 3). Nevertheless, α remained systematically underestimated even when the full phylogeny of 104 species was used (Fig. 3). In addition, the posterior distributions of α largely overlapped between datasets simulated under pure drift (BM) and weak stabilizing selection (OU with $t_{1/2}=1$), indicating limited power to distinguish between these evolutionary scenarios (Fig. 2). Varying levels of within-species variance had a minor impact on estimation accuracy and precision in our simulations with larger within-species variance slightly shifting to posterior estimates toward the prior distribution (Figs 2 and 3).

**Fig. 2.**
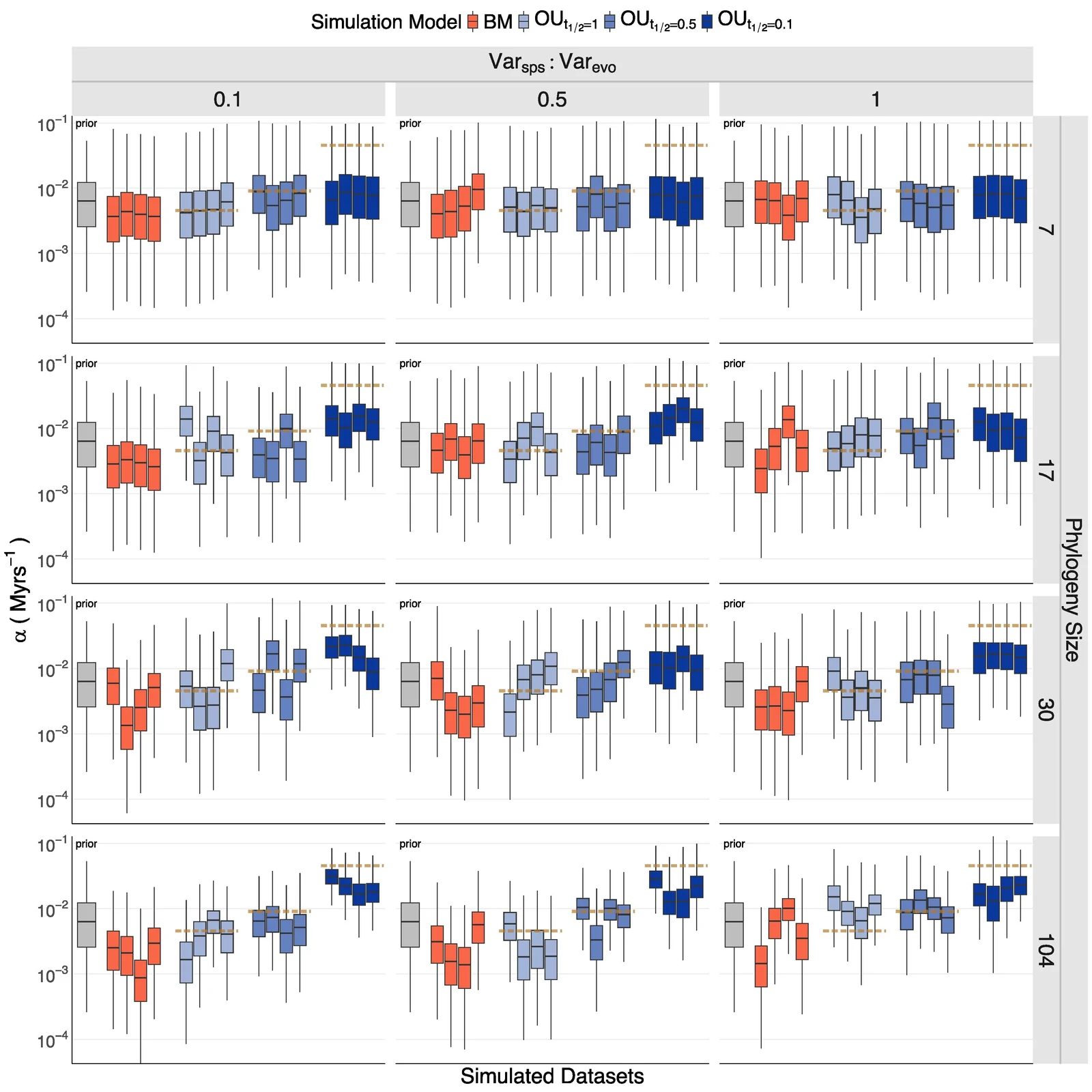
Posterior distribution of α of 4 random simulated datasets per settings. The models used for simulations (Brownian motion process, BM; and Ornstein–Uhlenbeck process, OU) are indicated by colors. Red denotes the simulations under the BM process, and blue represents the simulations under the OU process with different half-lives (light: 1, medium: 0.5, dark: 0.1, in the unit of total tree height). The values of α corresponding to the half-life in each model are shown with the dashed lines. The gray box on the left shows the prior distribution of α used in the inference. Each column shows the datasets simulated with three ratios of within-species variances to evolutionary variances (left: 0.1, middle: 0.5, right:1), and each row shows the size of the phylogeny used in the simulation (number of taxa: 7, 17, 30, and 104). The posterior distributions are shown as box plots, where the box represents the interquartile range, the horizontal line indicates the median, and the whiskers extend to the most extreme values within 1.5 times the interquartile range.

**Fig. 3.**
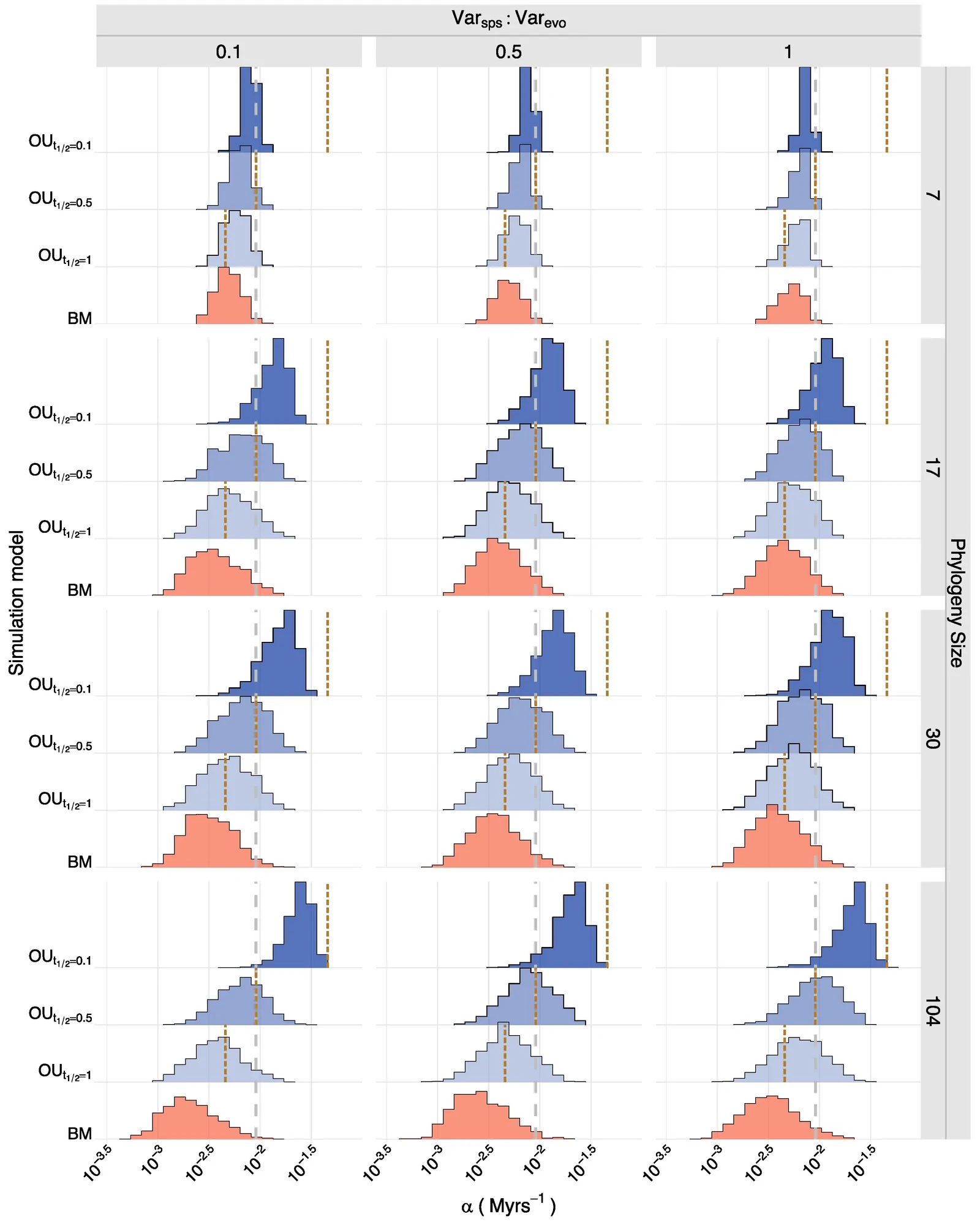
Distributions of posterior median estimates of α from simulated datasets. The models used for simulations (Brownian motion process, BM; and Ornstein–Uhlenbeck process, OU) are indicated by colors. Red denotes the simulations under the BM process, and blue represents the simulations under the OU process with different half-lives (light: 1, medium: 0.5, dark: 0.1, in the unit of total tree height). The gray dashed line is the prior median used for parameter estimation, and the brown dashed lines represent the corresponding value of α in each OU model. Each column shows the datasets simulated with three ratios of within-species variances to evolutionary variances (left: 0.1, middle: 0.5, right:1), and each row shows the size of the phylogeny used in the simulation (number of taxa: 7, 17, 30,104).

### Detecting Stabilizing Selection Through Model Selection

We performed model selection on each simulated dataset using RevBayes and EvoGeneX, to evaluate the power to detect stabilizing selection. The ability to correctly recover stabilizing selection was dependent on the phylogeny size, the true rate of selection, and the model selection threshold (Fig. 4). Under a relatively relaxed significance level (BF>3.2), the phylogeny with 17 species showed a clear increase in the proportion of datasets favoring the OU model. In particular, when the datasets were simulated under an OU model with strong selection ($t_{1/2}=0.1$), the correct model was supported in more than 80% of the cases. As a measure of false-positives, the datasets simulated under a BM model incorrectly favored the OU model in approximately 10%. Using a more stringent significance level (BF>20) reduced the false-positive rate; but only the phylogeny with 104 species achieved a high support for the correct model. Results obtained from EvoGeneX are generally similar to those obtained with the relaxed threshold (BF>3.2) in RevBayes.

**Fig. 4.**
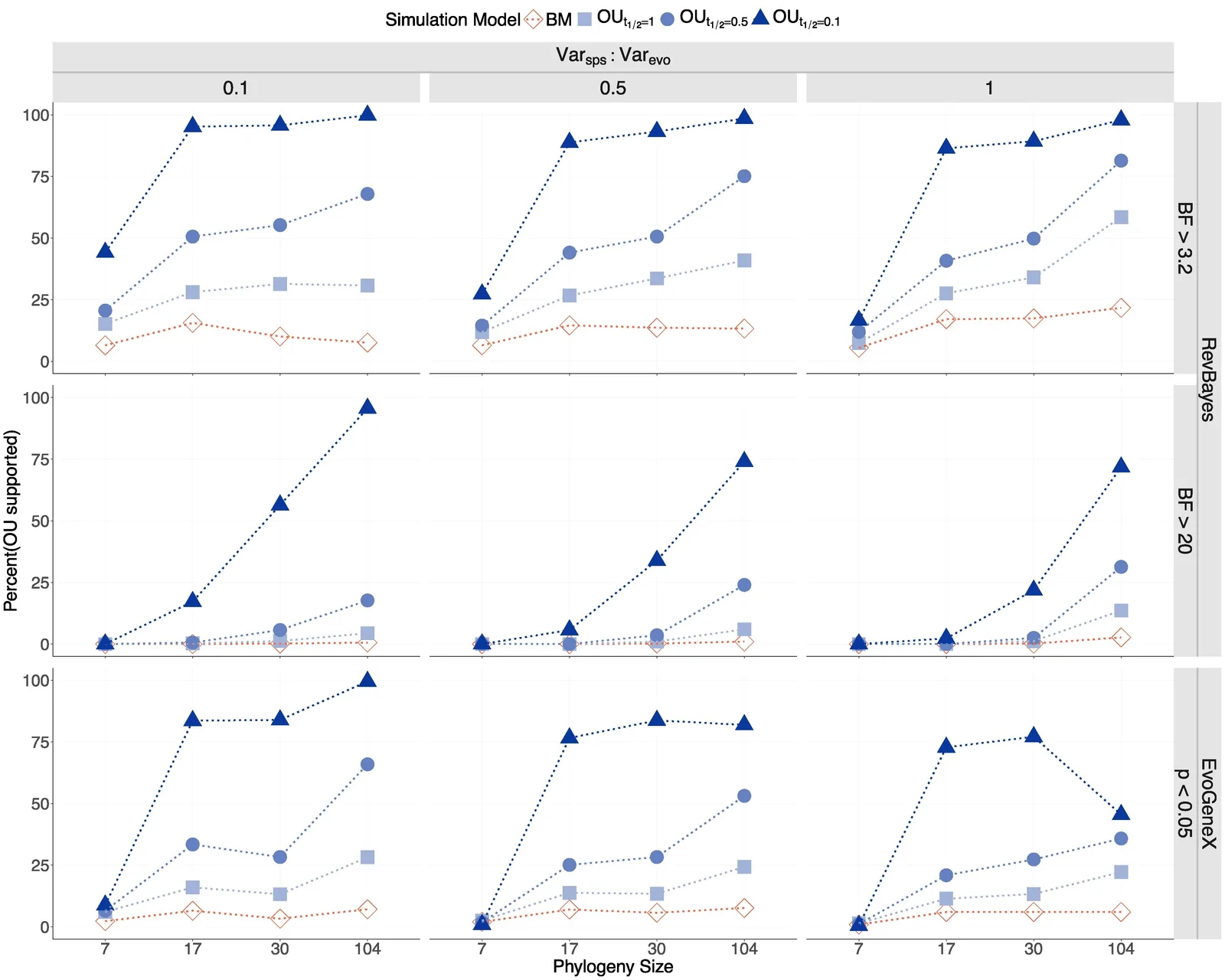
Percentage of simulated datasets supporting the Ornstein–Uhlenbeck process. Each dataset was simulated with three ratios of within-species variances to evolutionary variances (left: 0.1, middle: 0.5, right:1) on four phylogenies of equal height but different phylogeny sizes (*x*-axis). The models used for simulations (Brownian motion process, BM; and Ornstein–Uhlenbeck process, OU) are indicated by colors. The phylogenetic half-life of OU process was expressed in the unit of total tree height. Red denotes the simulations under the BM process, and blue represents the simulations under the OU process with different half-lives (light: 1, medium: 0.5, dark: 0.1). Each row shows the model selection results from two software packages with certain threshold, RevBayes (top: Bayesfactor[BF]>3.2, and middle: BF>20) and EvoGeneX (bottom: P<0.05).

Within-species variances had some influence on model selection, with the power to support the correct model generally decreasing as within-species variances increased. The false-positive rate slightly increased with larger within-species variances (Fig. 4). Surprisingly, for simulations with strong selection ($t_{1/2}=0.1$), we observed a decrease in the proportion of correctly supported datasets when using EvoGeneX for the large phylogeny (104 species) compared to the phylogeny with 30 species (Fig. 4), indicating that the implementation in EvoGeneX might be more sensitive to within-species variances.

## Discussion

We performed a simulation study based on an empirical phylogeny and realistic sampling strategies to evaluate the ability of the OU model to detect stabilizing selection in gene expression evolution. Our motivation was that many comparative studies aim to infer the dominant evolutionary mode of gene expression despite relying on datasets with a limited number of species with the additional complication of within-species variances (Fig. 1; Price et al. 2022). We therefore quantified how phylogeny size influences parameter estimation and the power to distinguish stabilizing selection from neutral drift. Our results show that, although accurately estimating the strength of selection (α) remains challenging, phylogenies with as few as 17 species can provide sufficient power to detect stabilizing selection under a relatively relaxed model selection threshold, if the underlying “true” selective force was sufficiently strong.

### Accuracy of Selection Strength Estimation

Neutral drift is represented when the selection strength α=0; conversely, stabilizing selection is present when α>0. However, how much larger than zero α must be to infer stabilizing selection is not straightforward. Our results show that when selection is weak (OU, $t_{1/2}=0.5$ and $t_{1/2}=1$) or absent (BM), the estimated values of α largely overlap across three models (Fig. 3), which indicates that estimates of α cannot serve as a good indicator of OU model to distinguish stabilizing selection when working with small phylogenies. Moreover, estimates of α could be biased, yet few studies have evaluated their accuracy. We found that the selection strength α is underestimated when selection is strong, especially with smaller phylogeny sizes ($t_{1/2}=0.1$ in Fig. 3). The ability to estimate the strength of selection α improves accurately with increasing phylogeny size, consistent with earlier studies (Beaulieu et al. 2012; Catalán et al. 2019; Chen et al. 2019). Furthermore, we explored alternative transformations of the selection strength α, ie the phylogenetic half-life $t_{1/2}$ and amount of variance reduced over time *ρ* (Cornuault 2022). Similarly to directly assessing the strength of selection α, neither $t_{1/2}$ nor *ρ* was able to indicate the existence of selection for small phylogenies and when the true selection was weak (Figs S1 and S2). Our results indicate that estimates of the selection strength *α* from small phylogenies should be interpreted with caution, and therefore detection of stabilizing selection might be limited.

### Detection of Stabilizing Selection by Model Selection

The influence of the phylogeny size on model selection is evident in our results. Under a relaxed significance level (BF>3.2), a phylogeny with 17 taxa is required to correctly identify stabilizing selection when the signal is strong. More than 30 taxa are required when adopting a more stringent significance level (BF>20) (see also Catalán et al. 2019).

Besides phylogeny size, other aspects have been shown to affect the power of model selection, e.g., the shape of the phylogeny (Cressler et al. 2015). Pal et al. (2023) argued that the evolutionary distance is more important in model selection, which we explored in our simulations with one species per selected genus. Despite this increased evolutionary distance, our results both using RevBayes and EvoGeneX showed that seven species are insufficient to statistically recover stabilizing selection (Fig. 4). Therefore, we recommend at least 17 taxa to reliably infer stabilizing selection in gene expression. For studies with few taxa, we recommend to perform a targeted simulation study as the power to detect stabilizing selection depends on additional factors such as the given shape of the phylogeny.

Moreover, our results showed that the power to correctly recover stabilizing selection depends on the strength of selection (see also Boettiger et al. 2012). In particular, weak selection (small but nonzero α) is inherently difficult to distinguish from neutral drift (α=0), resulting in uncertain estimates of α and limited power for model selection. From our simulations, we considered selection to be weak when the phylogenetic half-life was ≥0.5× the total tree height; at this half-life, gene expression is expected to move only 75% toward the optimum over the evolutionary history represented by the tree. This relatively weak signal makes it difficult to detect stabilizing selection, particularly with a limited number of taxa. Therefore, results indicating an inferred neutral drift or a weak stabilizing selection for small phylogenies should be viewed with caution. For example, Catalán et al. (2019) found the neutral drift explained gene expression data of more than 90% genes in *Heliconius* butterflies better than stabilizing selection, but using only five species (see also Dimayacyac et al. 2023). In contrast, we considered selection to be strong when the phylogenetic half-life was ≤0.1× the total tree height, such that gene expression is expected to reach approximately 99.9% of the optimum over the evolutionary history represented by the tree. Under such strong stabilizing selection, we found that a phylogeny of at least 17 species was sufficient to distinguish stabilizing selection from neutral drift. However, the observed among-species variation is determined not only by the strength of selection α but also by the evolutionary rate *σ*, which should therefore also be considered when interpreting the results.

### Implications for Modelling Gene Expression Evolution

Most studies exploring gene expression evolution still rely on datasets with a limited number of taxa (Fig. 1, Table S1). Our results on the lack of power to detect stabilizing selection for small phylogenies therefore raise important implications for how gene expression evolution should be modeled in practice.

Gene expression is often found to evolve under directional selection (e.g., Blekhman et al. 2008), which can be modeled by an Ornstein–Uhlenbeck process with multiple (branch-specific) optima (Butler and King 2004; Rohlfs and Nielsen 2015; Catalán et al. 2019; Chen et al. 2019; Pal et al. 2023). On the one hand, our results raise concerns about the feasibility to fit these more complex OU models if we cannot reliably infer the simpler OU model. On the other hand, distinguishing a single-optimum OU model from a OU model with two or more optima could be easier than estimating the strength of selection as the data are more informative about the optima (Cressler et al. 2015; Grabowski et al. 2023). More work is needed to establish our ability to detect directional selection.

Additionally, within-species variances have received increasing attention in studies on gene expression evolution using phylogenetic methods (Rohlfs et al. 2014; Catalán et al. 2019; Pal et al. 2023). Our results show that within-species variances play a lesser role in determining the power to detect stabilizing selection. However, smaller within-species variance can be an indicator of stabilizing selection itself (Rohlfs and Nielsen 2015), and the full power of this source of information is still underexplored.

Regarding the interpretation of the Bayes Factor (BF), our analyses used BF to quantify the relative support for stabilizing selection (OU) over neutral drift (BM). Thus, a larger BF indicates stronger evidence that gene expression evolution is better explained by stabilizing selection than by drift. We considered two thresholds to reflect different levels of evidence: BF>3.2 represents substantial support for stabilizing selection, whereas BF>20 represents strong support (Kass and Raftery 1995). The former threshold is approximately comparable to a conventional *P*-value of <0.05 under certain conditions (Schimmack 2015). Using these two thresholds allowed us to evaluate how the necessary phylogeny size changes with the strength of evidence required to conclude that gene expression evolution is under stabilizing selection.

## Conclusions

We performed a simulation study to evaluate the power to detect stabilizing selection compared to neutral drift in gene expression evolution across a range of phylogeny sizes, selection strengths and levels of within-species variance. Our results showed that random and unbiased within-species variances have a minor impact on both parameter estimation and model selection. When the true signal of stabilizing selection is sufficiently strong, the power to correctly reject a neutral drift model increases substantially with the phylogeny size. However, distinguishing weak selection from pure neutral drift remains challenging, even with phylogenies comprising >100 taxa. Consequently, we recommend that future studies of gene expression evolution can adopt a relatively relaxed model selection threshold (e.g., BF>3.2 or P<0.05) and include at least 17 taxa to detect stabilizing selection, although at the cost of a slightly inflated false-positive rate (Fig. 4). Finally, as the OU model is the foundation for further biological hypotheses, e.g., studying directional selection, more work, and discussion on the utility of the OU model as an underlying framework is required.

## Methods

### Modeling Gene Expression Evolution Across a Phylogeny

We assume that the mean expression level µ evolves along a phylogeny under either a BM or an OU process (Bedford and Hartl 2009; Rohlfs et al. 2014). Under the BM process, the mean expression level µ evolves under random drift with rate *σ*. Under the OU process, by contrast, the mean expression level µ is additionally pulled by a rate of selection α toward an optimum *θ*. The individual expression level $X_{ij}$ of sample *j* from species *i* was modeled as a normal distribution centered on the species mean expression level $\mu_{i}$, with a species-specific standard deviation $\epsilon_{i}$ as a parameter. The phylogenetic gene expression evolution models and their implementation in RevBayes were described in detail in Catalán et al. (2019).

### Data Simulation

Our goal was to explore how the choice of species sampling impacts the power to detect stabilizing selection in gene expression evolution. Our own research is focused on fireflies (Coleoptera: Lampyridae), and therefore we used our recent Lampyridae phylogeny with 104 extant species (Höhna et al. 2025) for data simulation. To evaluate the influence of phylogeny size, we extracted four trees reflecting empirical sampling strategies: (i) a tree containing one representative species from seven selected genera (seven species); (ii) a tree with multiple species within these seven selected genera (17 species); (iii) a tree including one species from each genus present in the phylogeny (30 species); and (iv) the whole phylogeny (104 species). All four extracted trees have the same tree-height and vary in the number of species.

We chose a range of selection strengths (α) to investigate the sensitivity to detect stabilizing selection. However, since the absolute value of α is difficult to interpret directly, we parameterize α based on the phylogenetic half-life $t_{1/2}$ (Equation 1), which represents the expected time for a trait to evolve halfway toward its optimum (Hansen 1997).


(1) $$t_{1/2}=\frac{\ln(2)}{\alpha }$$

The phylogenetic half-life is usually expressed in units of the total height of the phylogeny (Hansen et al. 2008; Cooper et al. 2016). Accordingly, in our simulations, we set $t_{1/2}$ to 1×, 0.5×, and 0.1× the total tree-height to represent weak to strong stabilizing selection and additionally α=0 (the BM process) as neutral drift.

We defined a series of within-species variances $\mathrm{Va}r_{\mathrm{sps}}$ relative to the evolutionary variance among species $\mathrm{Va}r_{\mathrm{evo}}$. For the BM process, the evolutionary variance corresponds to the expected variances accumulated over the total tree height *T*, whereas for the OU process, we used the stationary variance of the OU process as the measure of the evolutionary variances (Equation 2).


(2) $$\begin{array}{c} \mathrm{Va}r_{\mathrm{evo}}=\{\begin{array}{cc} \sigma^{2}T & \alpha =0,\mathrm{BM}\\ \sigma^{2}/2\alpha & \alpha >0,\mathrm{OU} \end{array} \end{array}$$

For each of the four phylogenies, we simulated 1,000 replicates of single-gene datasets under four models (the BM process and the OU process with three different phylogenetic half-lives) with three ratios of within-species to evolutionary variances. Data simulation was performed in RevBayes (Höhna et al. 2016). In total, we generated 48,000 datasets (Table 1).

**Table 1:** Summary of simulation settings

Simulation setting	
Phylogeny size	7, 17, 30, 104
Model	BM,
	OU with three different phylogenetic half-lives: 0.1×, 0.5×, 1× total tree height
$\mathrm{Va}r_{\mathrm{sps}}:\mathrm{Va}r_{\mathrm{evo}}$	0.1, 0.5, 1
	1,000 replicates per setting

### Parameter Estimation and Model Selection

We performed Bayesian inference under an OU model for each gene. Additionally, we performed Bayesian model selection between a BM model and an OU model based on Bayes Factors (BF, Kass and Raftery 1995). Analyses were conducted in RevBayes (Höhna et al. 2016). We assumed a fixed phylogeny identical to the tree used for data simulation. The prior distribution for α was set as an exponential distribution, with the mean corresponding to half-life equal to half of the total tree height. The prior distribution for the variance of each species was defined as an exponential distribution with a mean of 1. We ran four independent replicates for 100,000 iterations and parameters were sampled every five iterations. Convergence was checked using R package convenience (Fabreti and Höhna 2022).

Additionally, we conducted model selection analysis on the simulated datasets using EvoGeneX (Pal et al. 2023). We followed their framework for testing between neutral evolution (BM process) and constrained evolution (OU process with one common optimum). We then compared the outcomes from EvoGeneX with those from our implementation in RevBayes.

## Supplementary Material

evag266_Supplementary_Data

## Data Availability

The scripts and the original simulated data underlying this article are available in Zenodo at https://doi.org/10.5281/zenodo.18374738.
