# Supplementary material for: On the Number of Species Required to Detect Stabilizing Selection in Gene Expression Evolution
Source: Genome Biol Evol. 2026 Oct 24;18(11):evag266. doi: 10.1093/gbe/evag266 (PMC13767865; doi:10.1093/gbe/evag266)
Supplement: Supplementary file 1 [file evag266_supplementary_data.pdf]

On the number of species required to detect stabilizing selection in gene expression evolution

Supplementary Materials

Contents

Supplementary Figure S1 2
Supplementary Figure S2 3
Supplementary Table S1 4

Supplementary Figure S1

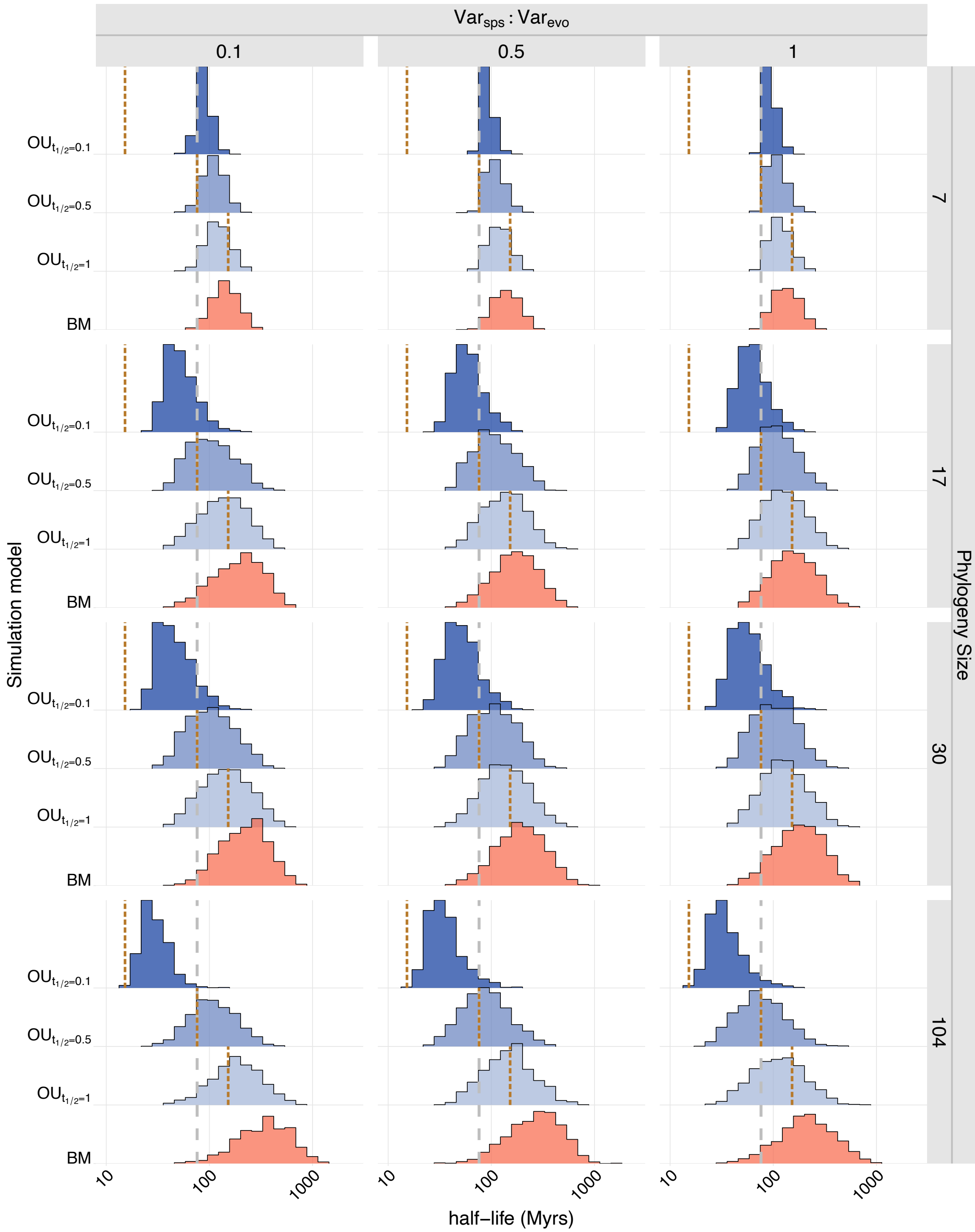

Figure S1: Distributions of posterior median estimates of phylogenetic half-life from simulated datasets. The models used for simulations (Brownian Motion process, BM; and Ornstein-Uhlenbeck process, OU) are indicated by colors. Red denotes the simulations under the BM process, and blue represents the simulations under the OU process with different half-lives (light: 1, medium: 0.5, dark: 0.1, in the unit of total tree height). The grey dashed line is the prior median used for parameter estimation, and the brown dashed lines represent the half-life in each OU model. Each column shows the datasets simulated with three ratios of within-species variances to evolutionary variances (left: 0.1, middle: 0.5, right:1), and each row shows the size of the phylogeny used in the simulation (number of tips: 7, 17, 30 ,104).

Supplementary Figure S2

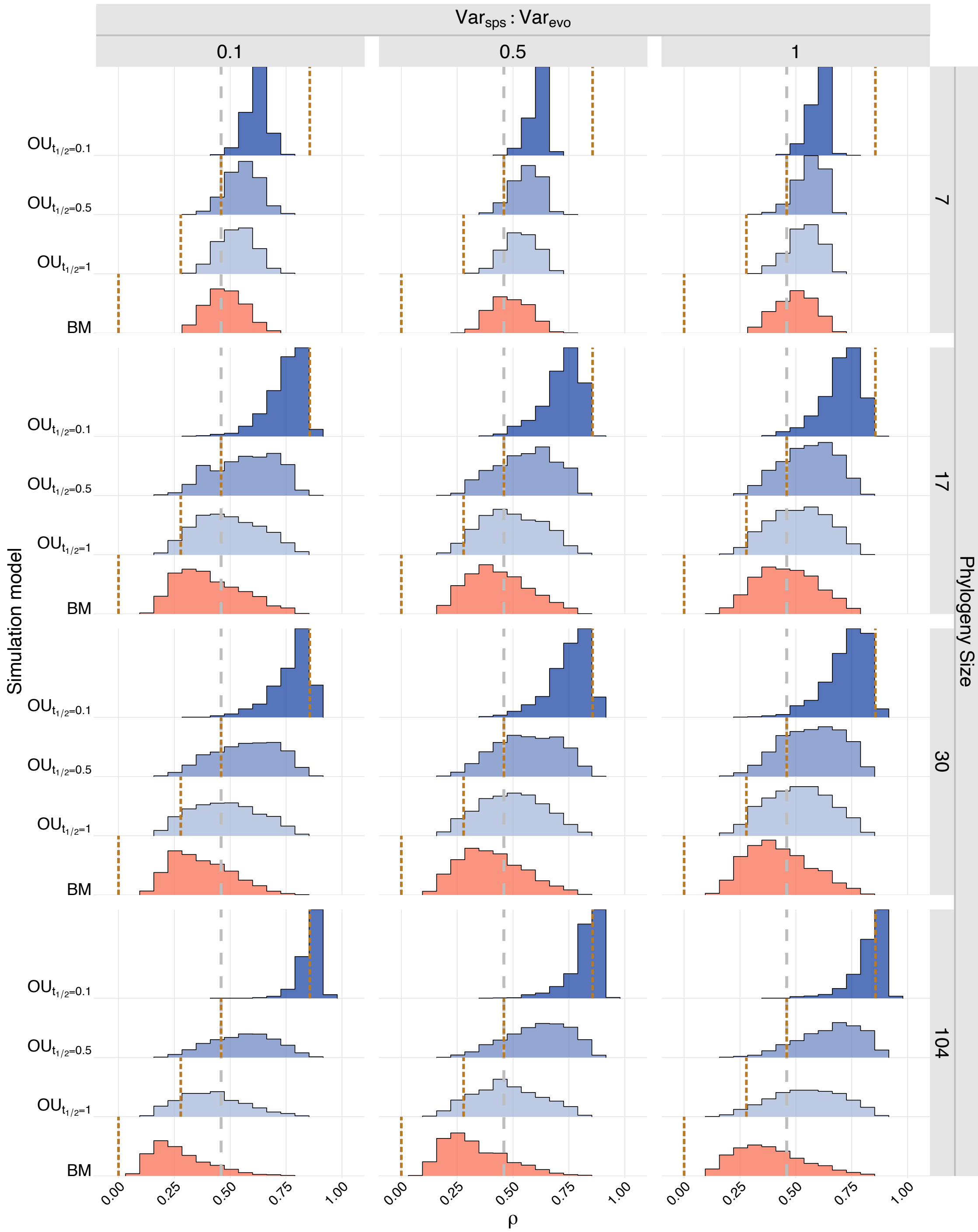

Figure S2: Distributions of posterior median estimates of ρ from simulated datasets. The models used for simulations (Brownian Motion process, BM; and Ornstein-Uhlenbeck process, OU) are indicated by colors. Red denotes the simulations under the BM process, and blue represents the simulations under the OU process with different half-lives (light: 1, medium: 0.5, dark: 0.1, in the unit of total tree height). The grey dashed line is the prior median used for parameter estimation, and the brown dashed lines represent the half-life in each OU model. Each column shows the datasets simulated with three ratios of within-species variances to evolutionary variances (left: 0.1, middle: 0.5, right:1), and each row shows the size of the phylogeny used in the simulation (number of tips: 7, 17, 30 ,104).

Supplementary Table S1

We surveyed comparative transcriptomic studies published between 2011 and 2026 that included gene expression data from at least three taxa. For each study, we recorded the number of taxa, the number of genes analyzed, the average number of biological samples per taxon, and the source of the gene expression data. Datasets were classified as newly generated, obtained from public datasets, or a combination of both.

Table S1: Summary of datasets used in previous studies of gene expression evolution. The table summarizes the datasets used in published studies, including the numbers of taxa, genes, and the average number of biological samples (or replicates) per taxon (range in parentheses). Taxa may correspond to species or intraspecific units (e.g., strains or populations). The "Source" column indicates whether the gene expression data were (1) newly generated, (2) obtained from public datasets, or (3) a combination of newly generated and public datasets.

Citation	# taxa	# genes	# samples/taxon	Taxonomic group	Source
Brawand et al. (2011)	10	5,636	2.2 (1-6)	Primates + outgroup	1
Cortez et al. (2014)	16	–	1.4 (1-6)	Mammals + birds	3
Brauer et al. (2017)	5	27,425	5.2 (4-6)	*Nannoperca australis*	1
Stern and Crandall (2018)	14	3,560	2.6 (1-3)	Freshwater crayfish	1
Chen et al. (2019)	17	10,899	2.3 (1-6)	Mammals	3
Assis (2019)	3	11,142	–	Grasses	2
Osborne et al. (2019)	8	26,972	6.1 (1-19)	*Howea* and relatives	3
Catalán et al. (2019)	5	2,393	9.8 (6-12)	*Heliconius*	1
Vegesna et al. (2020)	6	9	1.7 (1-4)	Apes	3
Bernal et al. (2020)	5	16,494	16.6 (14-20)	Coral reef fishes	1
Cope et al. (2020)	18	3,556	1.3 (1-3)	Fungi	2
Greenway et al. (2020)	20	5,000	5.9 (5-6)	Poeciliidae	1
Sandoval-Castillo et al. (2020)	3	34,815	6 (6)	Australian rainbowfish	1
Fukushima and Pollock (2020)	21	1,377	15.1 (1-255)	Vertebrates	2
El Taher et al. (2021)	74	31,824	6.0 (6-7)	Cichlids	1
Gillard et al. (2021)	7	10,154	3.8 (3-4)	Salmonids + outgroups	1
Avila-Magaña et al. (2021)	3	–	3 (3)	Caribbean corals	1
Tobler et al. (2021)	20	16,740	5.9 (5-6)	Poeciliids	1
Kopania et al. (2022)	4	23,164	8.5 (3-12)	Mice	1
Dimos et al. (2022)	5	4,277	5 (5)	Corals	1
Kang et al. (2022)	6	14,456	10 (7-12)	Coral reef fishes	1
Firneno Jr et al. (2022)	10	727	2.7 (2-3)	True toads	1
Pal et al. (2023)	9	8,591	4 (4)	*Drosophila*	1
Breeschoten et al. (2022)	5	8,709	3 (3)	Noctuid moths	1
Saavedra et al. (2023)	4	11,025	9 (4-13)	Marine clams	1
Beavers et al. (2023)	5	1,817	3.8 (1-5)	Corals	1
Kagan and Hejnol (2024)	43	5,612	–	Metazoa	3
Zhang et al. (2024)	4	–	3 (3)	*Daphnia*	1
Zancolli et al. (2025)	12	2,588	2.9 (1-5)	Gastropods	1
Lipshutz et al. (2025)	10	10,672	6 (6)	Songbirds	1
Thomas et al. (2025)	16	6,496	6.3 (3-8)	Mammals	3
Mead et al. (2025)	6	16,736	3 (3)	Oaks	1
Chalopin et al. (2026)	33	11,437	3.5 (1-6)	Rodents	3
Glaser-Schmitt and Parsch (2026)	6	9,016	6.2 (5-8)	*Drosophila*	1

References

R. Assis. Lineage-specific expression divergence in grasses is associated with male reproduction, host-pathogen defense, and domestication. *Genome biology and evolution*, 11(1):207–219, 2019.

V. Avila-Magaña, B. Kamel, M. DeSalvo, K. Gómez-Campo, S. Enríquez, H. Kitano, R. V. Rohlfs, R. Iglesias-Prieto, and M. Medina. Elucidating gene expression adaptation of phylogenetically divergent coral holobionts under heat stress. *Nature Communications*, 12(1):5731, 2021.

K. M. Beavers, E. W. Van Buren, A. M. Rossin, M. A. Emery, A. J. Veglia, C. E. Karrick, N. J. MacKnight, B. A. Dimos, S. S. Meiling, T. B. Smith, et al. Stony coral tissue loss disease induces transcriptional signatures of in situ degradation of dysfunctional symbiodiniaceae. *Nature Communications*, 14(1):2915, 2023.

M. A. Bernal, C. Schunter, R. Lehmann, D. J. Lightfoot, B. J. Allan, H. D. Veilleux, J. L. Rummer, P. L. Munday, and T. Ravasi. Species-specific molecular responses of wild coral reef fishes during a marine heatwave. *Science Advances*, 6(12):eaay3423, 2020.

C. J. Brauer, P. J. Unmack, and L. B. Beheregaray. Comparative ecological transcriptomics and the contribution of gene expression to the evolutionary potential of a threatened fish. *Molecular Ecology*, 26(24): 6841–6856, 2017.

D. Brawand, M. Soumillon, A. Necsulea, P. Julien, G. Csárdi, P. Harrigan, M. Weier, A. Liechti, A. Aximu-Petri, M. Kircher, et al. The evolution of gene expression levels in mammalian organs. *Nature*, 478(7369): 343–348, 2011.

T. Breeschoten, M. E. Schranz, E. H. Poelman, and S. Simon. Family dinner: Transcriptional plasticity of five noctuidae (lepidoptera) feeding on three host plant species. *Ecology and Evolution*, 12(9):e9258, 2022.

A. Catalán, A. D. Briscoe, and S. Höhna. Drift and directional selection are the evolutionary forces driving gene expression divergence in eye and brain tissue of heliconius butterflies. *Genetics*, 213(2):581–594, 2019.

D. Chalopin, C. Rey, J. Ganofsky, J. Blin, P. Chevret, M. Mouginot, L. Guéguen, B. Boussau, S. Pantalacci, and M. Sémon. Degrees of convergent evolution in rodent adaptations to arid environments. *Genome Research*, 36(3):472–486, 2026.

J. Chen, R. Swofford, J. Johnson, B. B. Cummings, N. Rogel, K. Lindblad-Toh, W. Haerty, F. d. Palma, and A. Regev. A quantitative framework for characterizing the evolutionary history of mammalian gene expression. *Genome Research*, 29(1):53–63, 2019.

A. L. Cope, B. C. O'Meara, and M. A. Gilchrist. Gene expression of functionally-related genes coevolves across fungal species: detecting coevolution of gene expression using phylogenetic comparative methods. *BMC genomics*, 21(1):370, 2020.

D. Cortez, R. Marin, D. Toledo-Flores, L. Froidevaux, A. Liechti, P. D. Waters, F. Grützner, and H. Kaessmann. Origins and functional evolution of y chromosomes across mammals. *Nature*, 508(7497):488–493, 2014.

B. Dimos, M. Emery, K. Beavers, N. MacKnight, M. Brandt, J. Demuth, and L. Mydlarz. Adaptive variation in homologue number within transcript families promotes expression divergence in reef-building coral. *Molecular ecology*, 31(9):2594–2610, 2022.

A. El Taher, A. Böhne, N. Boileau, F. Ronco, A. Indermaur, L. Widmer, and W. Salzburger. Gene expression dynamics during rapid organismal diversification in african cichlid fishes. *Nature Ecology & Evolution*, 5 (2):243–250, 2021.

T. J. Firneno Jr, B. Ramesh, J. A. Maldonado, A. I. Hernandez-Briones, A. H. Emery, C. E. Roelke, and M. K. Fujita. Transcriptomic analysis reveals potential candidate pathways and genes involved in toxin biosynthesis in true toads. *Journal of Heredity*, 113(3):311–324, 2022.

K. Fukushima and D. D. Pollock. Amalgamated cross-species transcriptomes reveal organ-specific propensity in gene expression evolution. *Nature communications*, 11(1):4459, 2020.

G. B. Gillard, L. Grønvold, L. L. Røsæg, M. M. Holen, Ø. Monsen, B. F. Koop, E. B. Rondeau, M. K. Gundappa, J. Mendoza, D. J. Macqueen, et al. Comparative regulomics supports pervasive selection on gene dosage following whole genome duplication. *Genome Biology*, 22(1):103, 2021.

A. Glaser-Schmitt and J. Parsch. Conservation and divergence of sex-biased gene expression across 50 million years of drosophila evolution. *Molecular Biology and Evolution*, 43(4):msag081, 2026.

R. Greenway, N. Barts, C. Henpita, A. P. Brown, L. Arias Rodriguez, C. M. Rodríguez Peña, S. Arndt, G. Y. Lau, M. P. Murphy, L. Wu, et al. Convergent evolution of conserved mitochondrial pathways underlies repeated adaptation to extreme environments. *Proceedings of the National Academy of Sciences*, 117(28): 16424–16430, 2020.

F. Kagan and A. Hejnol. Comparative analysis of maternal gene expression patterns unravels evolutionary signatures across reproductive modes. *Molecular Biology and Evolution*, 41(5):msae081, 2024.

J. Kang, I. Nagelkerken, J. L. Rummer, R. Rodolfo-Metalpa, P. L. Munday, T. Ravasi, and C. Schunter. Rapid evolution fuels transcriptional plasticity to ocean acidification. *Global Change Biology*, 28(9):3007–3022, 2022.

E. E. Kopania, E. L. Larson, C. Callahan, S. Keeble, and J. M. Good. Molecular evolution across mouse spermatogenesis. *Molecular Biology and Evolution*, 39(2):msac023, 2022.

S. E. Lipshutz, M. S. Hibbins, A. B. Bentz, A. M. Buechlein, T. A. Empson, E. M. George, M. E. Hauber, D. B. Rusch, W. M. Schelsky, Q. K. Thomas, et al. Repeated behavioural evolution is associated with convergence of gene expression in cavity-nesting songbirds. *Nature Ecology & Evolution*, 9(5):845–856, 2025.

A. Mead, C. D. Medeiros, M. E. Ochoa, L. Sack, and V. L. Sork. Drought tolerance is associated with constitutive gene expression, not plasticity, across california oak species. *bioRxiv*, pages 2025–08, 2025.

O. G. Osborne, A. Ciezarek, T. Wilson, D. Crayn, I. Hutton, W. J. Baker, C. G. Turnbull, and V. Savolainen. Speciation in howea palms occurred in sympatry, was preceded by ancestral admixture, and was associated with edaphic and phenological adaptation. *Molecular Biology and Evolution*, 36(12):2682–2697, 2019.

S. Pal, B. Oliver, and T. M. Przytycka. Stochastic modeling of gene expression evolution uncovers tissue- and sex-specific properties of expression evolution in the drosophila genus. *Journal of Computational Biology*, 30(1):21–40, 2023.

C. Saavedra, M. Milan, R. B. Leite, D. Cordero, T. Patarnello, M. L. Cancela, and L. Bargelloni. Transcriptional profiling of populations in the clam ruditapes decussatus suggests genetically determined differentiation in gene expression along parallel temperature gradients and between races of the atlantic ocean and west mediterranean sea. *Fishes*, 8(4):203, 2023.

J. Sandoval-Castillo, K. Gates, C. J. Brauer, S. Smith, L. Bernatchez, and L. B. Beheregaray. Adaptation of plasticity to projected maximum temperatures and across climatically defined bioregions. *Proceedings of the National Academy of Sciences*, 117(29):17112–17121, 2020.

D. B. Stern and K. A. Crandall. The evolution of gene expression underlying vision loss in cave animals. *Molecular biology and evolution*, 35(8):2005–2014, 2018.

W. R. Thomas, T. Richter, E. T. O'Neil, C. Baldoni, A. Corthals, D. von Elverfeldt, J. D. Nieland, D. Dechmann, R. Hunter, and L. M. Davalos. Seasonal and comparative evidence of adaptive gene expression in mammalian brain size plasticity. *Elife*, 13:RP100788, 2025.

M. Tobler, R. Greenway, and J. L. Kelley. Ecology drives the degree of convergence in the gene expression of extremophile fishes. *bioRxiv*, pages 2021–12, 2021.

R. Vegesna, M. Tomaszkiewicz, O. A. Ryder, R. Campos-Sánchez, P. Medvedev, M. DeGiorgio, and K. D. Makova. Ampliconic genes on the great ape y chromosomes: rapid evolution of copy number but conservation of expression levels. *Genome biology and evolution*, 12(6):842–859, 2020.

G. Zancolli, M. V. Modica, N. Puillandre, Y. Kantor, A. Barua, G. Campli, and M. Robinson-Rechavi. Redistribution of ancestral functions underlies the evolution of venom production in marine predatory snails. *Molecular biology and evolution*, 42(5):msaf095, 2025.

X. Zhang, L. Wang, Z. Deng, D. Blair, W. Hu, and M. Yin. Adaptation of a keystone aquatic crustacean to cold temperatures on the qinghai–tibetan plateau. *Limnology and Oceanography*, 69(11):2651–2667, 2024.
